# Structural Basis for Targeting the Bifunctional Enzyme ArnA

**DOI:** 10.3390/biom15111594

**Published:** 2025-11-13

**Authors:** Xinyu Liu, Ruochen Yang, Libang Ren, Tong Li, Yanrong Li, Zhihua Yan, Yanrong Gao, Mingqi Yang, Jiazhi Li

**Affiliations:** 1 Institutes of Biomedical Sciences, Inner Mongolia University, Hohhot 010020, China; 32308280@mail.imu.edu.cn (X.L.); 32492012@mail.imu.edu.cn (R.Y.); 22592009@mail.imu.edu.cn (L.R.); 32492023@mail.imu.edu.cn (Y.L.); 32592010@mail.imu.edu.cn (Z.Y.); 22308054@mail.imu.edu.cn (Y.G.); 2 School of Life Sciences, Inner Mongolia University, Hohhot 010020, China; 0222121363@mail.imu.edu.cn (T.L.); 0232121446@mail.imu.edu.cn (M.Y.)

**Keywords:** ArnA, L-Ara4N modification, structure, peptide inhibitor

## Abstract

Polymyxin antibiotics are often the last line of defense against multidrug-resistant Gram-negative pathogens. A key resistance mechanism involves the addition of 4-amino-4-deoxy-L-arabinose (L-Ara4N) to lipid A, mediated by the bifunctional enzyme ArnA. However, the evolutionary rationale and structural basis for ArnA’s domain fusion, hexameric assembly, and catalytic coordination remain mechanistically unresolved. Here, we integrate evolutionary genomics, high-resolution cryo-electron microscopy (cryo-EM), and computational protein design to provide a comprehensive mechanistic analysis of ArnA. Our evolutionary analysis reveals that the dehydrogenase (DH) and formyltransferase (TF) domains evolved independently and were selectively fused in Gammaproteobacteria, suggesting an adaptive advantage. A 2.89 Å cryo-EM structure of apo-ArnA resolves the flexible interdomain linker and reveals a DH-driven hexameric architecture essential for enzymatic activity. 3D variability analysis captures intrinsic conformational dynamics, indicating a molecular switch that may coordinate sequential catalysis and substrate channeling. Structure-based peptide inhibitors targeting the hexamerization and predicted ArnA–ArnB interaction interfaces were computationally designed, offering a novel strategy for disrupting L-Ara4N biosynthesis. These findings illuminate a previously uncharacterized structural mechanism of antimicrobial resistance and lay the groundwork for therapeutic intervention.

## 1. Introduction

Bacterial antimicrobial resistance (AMR) has become one of the major global public health threats of the 21st century. The Review on Antimicrobial Resistance, commissioned by the UK government, warned that without effective control, AMR could cause 10 million deaths annually by 2050 [1,2]. Empirical studies from 2019 demonstrated that several multidrug-resistant (MDR) bacteria, specifically, *Escherichia coli*, *Staphylococcus aureus*, *Klebsiella pneumoniae*, *Streptococcus pneumoniae*, *Acinetobacter baumannii*, and *Pseudomonas aeruginosa*, each contributed to more than 250,000 AMR-attributable deaths. Among these, *E. coli* was identified as the leading pathogen responsible for deaths attributable to AMR [3,4]. Comprehensive assessments confirm that bacterial AMR undoubtedly represents a significant challenge in global health.

Cationic Antimicrobial Peptides (CAMPs) are a class of antimicrobial peptides characterized by cationic groups and hydrophobic domains. Polymyxins, such as Polymyxin B and Polymyxin E (Colistin), which belong to a group of Non-ribosomal CAMPs, exert their mechanism of action through electrostatic interactions with the lipid A component of lipopolysaccharide (LPS) in the outer membrane of Gram-negative bacteria. This binding disrupts membrane integrity, leading to the formation of transmembrane pores and ultimately resulting in the leakage of intracellular contents and cell lysis due to osmotic imbalance [1,4,5,6,7]. Polymyxins have become critical antibiotics in combating multidrug-resistant Gram-negative bacteria and demonstrate significant value in clinical applications [1,7].

Gram-negative bacteria have evolved diverse mechanisms to counteract the bactericidal activity of polymyxins. A key resistance strategy involves modifying the overall negatively charged lipid A component of their lipopolysaccharide (LPS) by adding 4-amino-4-deoxy-L-arabinose (L-Ara4N) to its phosphate groups [8,9]. This modification neutralizes the net negative charge of lipid A, thereby significantly reducing its electrostatic binding affinity for cationic polymyxin molecules such as polymyxin E. This mechanism not only confers resistance to polymyxin antibiotics but also constitutes an essential basis for bacterial evasion of host innate immune defenses mediated by cationic antimicrobial peptides (CAMPs) [8,9,10].

Gram-negative bacteria utilize an enzymatic system encoded by the *pmrE* (*ugd*) gene and the *arnBCADTEF* operon to accomplish the complete modification pathway of L-Ara4N (Figure S1) [8,11,12]. This pathway begins with UDP-glucose (UDP-Glc), which is oxidized to UDP-glucuronic acid (UDP-GlcA) by the action of PmrE. UDP-GlcA subsequently undergoes oxidative decarboxylation catalyzed by the dehydrogenase domain (ArnA_DH) of ArnA, a bifunctional protein that harbors two distinct enzymatic domains, yielding UDP-4-keto-arabinose (UDP-Ara4O) [11,13,14]. ArnB then catalyzes a transamination reaction, using glutamate as the amino donor, to produce UDP-4-amino-4-deoxy-L-arabinose (UDP-L-Ara4N) [15]. Subsequently, UDP-L-Ara4N is formylated in an N-formyltetrahydrofolate-dependent reaction catalyzed by the formyltransferase domain (ArnA_TF) of ArnA, forming UDP-L-Ara4FN. This intermediate is then transferred by ArnC, an inner membrane-integrated protein with undecaprenyl phosphate-4-deoxy-4-formamido-L-arabinose transferase activity, to yield undecaprenyl phosphate-4-deoxy-4-formamido-L-arabinose (Und-P-L-Ara4FN) [11]. Next, the deformylase ArnD hydrolyzes Und-P-L-Ara4FN to form undecaprenyl phosphate-4-amino-4-deoxy-L-arabinose (Und-P-L-Ara4N). After transport to the periplasmic side of the membrane, the L-Ara4N moiety is finally transferred to the 4′-phosphate of lipid A by the action of ArnT [11,16,17,18].

Given the crucial role of L-Ara4N modification in polymyxin resistance, key enzymes in its biosynthetic pathway have become a significant focus of research. Among them, ArnA has emerged as a key enzyme due to its bifunctional nature and central position in the pathway [13,15,19]. Crystallographic studies revealed that full-length ArnA assembles into a homohexamer. Compared to ligand-free structures, both the carboxylase and formyltransferase domains undergo conformational changes in their substrate-binding pockets upon ligand binding, with key catalytic residues identified within the active sites. These studies also demonstrated that the conformational flexibility of ArnA_DH is a unique mechanistic feature of the bacterial Ara4N modification pathway and proposed a catalytic mechanism involving substrate binding and product release [19,20,21,22]. Cryo-EM studies have observed a tetrameric state of ArnA, suggesting a dynamic equilibrium between hexamers and tetramers. Significant relative displacement (8–16 Å) between the carboxylase and formyltransferase domains results in a more compact conformation. It is proposed that such reversible oligomeric switching and dynamic conformational changes may promote functional coordination between ArnA and downstream enzymes, such as ArnB and ArnC [23].

Although previous studies have improved our understanding of ArnA, several key questions remain unresolved, including the functional significance of its bifunctional domain architecture, the mechanism and role of its hexameric assembly, and the coordination between its two catalytic domains. In addition, structure-based efforts to develop effective inhibitors have so far yielded limited results. In this study, we combined evolutionary, structural, and computational analyses to investigate the organization and catalytic coordination of ArnA. These findings deepen our mechanistic understanding and lay the groundwork for future therapeutic strategies against polymyxin resistance.

## 2. Materials and Methods

### 2.1. Identification and Phylogenetic Analysis of TF-DH Fusion Proteins

The dataset comprised proteomes from 1105 representative bacterial species, 123 representative archaeal species, and 648 representative eukaryotic species, sourced from the NCBI and Ensembl databases, respectively. Taxonomic classification for all species was performed using the Genome Taxonomy Database (GTDB) release R226 [24]. BLASTP (version 2.16.0) was employed for homology searches (E-value < 1 × 10^−5^), using the TF and DH domain sequences from *Escherichia coli* ArnA protein (UniProt ID: P77398) as queries for reciprocal BLAST. To achieve clear phylogenetic visualization while maintaining comprehensive taxonomic representation, we selected 311 representative species from the 1299 species harboring TF-DH homologous proteins. Representative species were selected following these principles to maximize taxonomic diversity: (i) prioritizing retention of species with fusion proteins; (ii) sampling from diverse taxonomic lineages to ensure species diversity. The final dataset comprised 311 species, encompassing 236 genera, 147 families, 77 orders, 9 classes, 5 phyla, and 2 domains.

To quantitatively assess the enrichment patterns of TF-DH domain fusion across different taxonomic levels, we performed hierarchical statistical analyses on 1299 species (including 877 bacteria, 391 eukaryotic, and 31 archaeal species). At each taxonomic level, we used two-tailed Fisher’s exact tests to compare differences in domain fusion frequencies between the target and control groups. We calculated odds ratios (ORs) to quantify the magnitude of enrichment. Specifically, comparisons were conducted at the domain level (Bacteria vs. Eukarya and Archaea); at the phylum level (Pseudomonadota vs. 51 other bacterial phyla); at the class level (Gammaproteobacteria vs. four different classes within Pseudomonadota: Betaproteobacteria, Alphaproteobacteria, Acidithiobacillia, and Candidatus Mariprofundia); and at the order level, Enterobacterales vs. the other 13 orders within Gammaproteobacteria. Given four primary independent tests (domain level, phylum level, class level, and order level tests), Bonferroni correction for multiple testing was applied with an adjusted significance threshold of α = 0.0125 (0.05/4). All statistical analyses were performed using base R statistical functions (R v4.5.1).

TF and DH domain homologs identified by BLAST were subjected to multiple sequence alignment separately using the MUSCLE algorithm in MEGA software (version 12), and the alignments of the two domains were then concatenated. Phylogenetic trees were constructed using the Maximum Likelihood method in MEGA12 with the JTT (Jones-Taylor-Thornton) model and 1000 ultrafast bootstrap replicates to assess branch support. Trees were visualized using the ggtree package (v3.16.3) in R (v4.5.1) and displayed in fan layout. Three concentric rings were shown to represent taxonomic classifications at the domain, class, and order levels (from inner to outer).

### 2.2. Molecular Cloning, Protein Expression and Purification

The genes encoding *E. coli* ArnA (UniProt: P77398) were amplified by PCR from *E. coli* genomic DNA. *E. coli* ArnA was amplified using forward primer 5′-CAGGATCCGAATTCGATGAAAACCGTCGTTTTTGCCTACCACG and reverse primer 5′-GCACCGTTGATCTTACGGATAAACCATCATGAAAGCTTGCGGCCGCATAA. The PCR products were gel-purified and ligated into a pETduet vector featuring an N-terminal His×6 tag.

The recombinant plasmid for ArnA expression was transformed into *E. coli* Rosetta (DE3) cells (Novagen, Darmstadt, Germany) cultured in Luria-Bertani (LB) medium containing 50 µg mL^−1^ ampicillin at 37 °C. Protein expression was induced at 16 °C with 0.1 mM Isopropyl-β-D-thiogalactopyranoside (IPTG) when an OD_600_ of 0.6–0.8 was reached. Cells were collected after overnight induction (~18 h) by centrifugation at 4000 rpm for 15 min using a Beckman JLA 8.1000 rotor and resuspended in lysis buffer (50 mM Tris-HCl, pH 7.5, 150 mM NaCl, 5% glycerol, 5 mM β-mercaptoethanol). After sonication, the supernatant was collected by centrifugation at 18,000 rpm for 60 min (4 °C) using a Beckman JA-30.50 Ti rotor. The clarified lysate was loaded onto a Ni^2+^-NTA agarose column that had been pre-equilibrated with 30 column volumes (CVs) of Ni^2+^-NTA wash buffer (50 mM Tris-HCl, pH 7.5, 150 mM NaCl). The column was washed with 5 CV of wash buffer (50 mM Tris-HCl, pH 7.5, 150 mM NaCl, 20 mM imidazole). Protein was eluted using a linear gradient of elution buffer (50 mM Tris-HCl, pH 7.5, 150 mM NaCl, 50–200 mM imidazole). Fractions containing ArnA protein, as identified by SDS–PAGE, were pooled. The NaCl concentration was diluted to 50 mM using dilution buffer (50 mM Tris-HCl, pH 7.5), and the sample was loaded onto an anion exchange chromatography column (HiTrap Q HP, Cytiva, Sigma-Aldrich, St. Louis, USA) for further purification using a linear NaCl gradient. High-purity ArnA fractions were pooled, concentrated, and further purified using a gel-filtration chromatography column (Superdex 200 increase 10/300 GL, Cytiva, Sigma-Aldrich) in buffer containing 50 mM Tris-HCl, 150 mM NaCl, and 1 mM Tris(2-carboxyethyl) phosphine (TCEP). ArnA eluted at a volume of 9.9 mL. Freshly purified protein was collected for cryo-EM grid preparation.

### 2.3. Cryo-EM Grid Preparation

Five microliters of sample at 0.7 mg mL^−1^ were applied to a glow-discharged Quantifoil R1.2/1.3 400 mesh copper grid (Electron Microscopy Sciences, Hatfield, PA, USA), blotted for 7 s in 100% humidity at 4 °C, and plunge-frozen in liquid ethane using an FEI Vitrobot Mark IV (Thermo Fisher Scientific, Waltham, MA, USA). All grids were screened using Thermo Fisher Talos L120C and Talos F200C microscopes (Center for Biological Imaging, Institute of Biophysics, Chinese Academy of Sciences, Beijing, China). For the ArnA sample applied onto graphene, 2681 and 3882 micrographs were collected using a 300 kV Titan Krios microscope (Thermo Fisher) equipped with a Falcon III, Ceta, and BioQuantum K2 camera, with a pixel size of 0.4 Å and a spherical aberration of 2.7 mm, and a total electron dose of 60 e^−^ Å^−2^ s^−1^. For ArnA without graphene, 4407 micrographs were collected using a 300 kV Titan Krios microscope (Thermo Fisher) equipped with a Falcon III and K3 camera using similar parameters.

### 2.4. Cryo-EM Data Processing

For the initial dataset of ArnA without graphene, 4407 movie stacks were collected. Patch Motion correction and patch CTF Estimation were performed using cryoSPARC [25]. Micrographs with poor CTF estimates, significant drift, or ice contamination were discarded. Initial particle picking from selected micrographs was performed using Blob Picker in cryoSPARC. 2D classification was used to generate templates for Topaz training. Subsequent particle extraction was performed using Topaz Extract with the optimized model from Topaz Train (v0.2.5). After two rounds of 2D classification to remove junk particles, 420,463 particles were selected for Ab-initio Reconstruction and Heterogeneous Refinement. The best class, containing 34% of the particles, was chosen for Non-uniform Refinement or Homogeneous Refinement, resulting in a map with a nominal resolution of 3.25 Å. Further processing, including Global CTF Refinement, Local CTF Refinement, and Sharpening Tools, resulted in a map with a resolution of 3.04 Å. However, this reconstruction exhibited a strong preferred orientation (Figure S2). All three-dimensional reconstructions were carried out using C1 symmetry.

To mitigate the preferred orientation, two additional datasets of ArnA applied onto graphene were collected, totaling 6563 movie stacks. Motion correction and CTF Estimation were performed as above. Selected micrographs were subjected to initial particle picking using Blob Picker in cryoSPARC. 2D classes from these particles were used for Topaz training. Particle extraction was then performed using the Topaz Extract software. After two rounds of 2D Classification, 892,305 particles were selected for Ab-initio Reconstruction and Heterogeneous Refinement. The best subset of particles (53%) was chosen for Non-uniform Refinement or Homogeneous Refinement, resulting in an initial map at a resolution of 2.91 Å. Subsequent Global CTF Refinement, Local CTF Refinement, and Sharpening resulted in a final map at 2.89 Å resolution (Figure S3). All three-dimensional reconstructions were carried out using C1 symmetry.

### 2.5. Model Building and Refinement

An initial model of ArnA was predicted using AlphaFold3 and then refined to fit the 2.89 Å cryo-EM map of the ArnA hexamer using ChimeraX (version 1.8) [26]. Manual adjustments were performed using Coot to yield the final atomic model [27]. Real-space refinement was performed against the cryo-EM density map, incorporating secondary structure and geometry restraints, using PHENIX (version 1.21.1) [28].

3D variability analysis was performed in cryoSPARC, and the resulting density variations were visualized using Chimera (version 1.18) [29]. Model building for the starting and ending states, based on the 3D variability analysis, followed the same procedure described above. All structural figures were generated using PyMOL (version 2.4), Chimera, and ChimeraX.

### 2.6. In Vitro Activity Assay of ArnA Mutants

Based on our analysis of the interaction interfaces in the ArnA cryo-EM structure, we designed a series of point mutants targeting the DH-DH interface, including ArnA(R418A), ArnA(R472A), ArnA(Y477A), ArnA(K480A), ArnA(E407A_E411A), ArnA(E407A_E411A_R472A), and ArnA(E407A_E411A_K480A). For comparison, mutants targeting other hexamerization interfaces were also constructed: ArnA(Q291A), ArnA(R303A), ArnA(K381A), ArnA(Q291A_R303A), and ArnA(Q291A_R303A_K381A). An active-site mutant, ArnA(N492A), previously shown to be inactive, was included as a negative control [21]. All mutants were expressed and purified.

Notably, during purification, three mutants, ArnA(Q291A_R303A), ArnA(Q291A_R303A_K381A), and ArnA(E407A_E411A_R472A), eluted as two distinct peaks in size-exclusion chromatography (SEC). SDS-PAGE analysis confirmed that both peaks contained full-length ArnA protein.

To assess the impact of hexamerization on catalytic activity, we performed in vitro dehydrogenase assays on WT ArnA, all mutants, and the SEC-resolved peaks from the oligomeric state variants. Reactions (50 µL) contained NAD^+^ at a final concentration of 3 mM, UDP-GlcA at 1.5 mM, and protein at 1 µM. The assays were conducted in 96-well plates at 37 °C. Absorbance at 340 nm was monitored using a microplate reader (Molecular Devices SpectraMax ID3), with readings taken every 30 s over 20 min. Data were processed in Prism, where the linear range was identified and used to calculate the reaction velocity (nmol min^−1^) for graphical representation.

### 2.7. Protein Design Using RFdiffusion, ProteinMPNN, and AlphaFold3

During the initial design phase of peptides aimed at inhibiting ArnA hexamer formation, residues 30–75 of the TF domain of ArnA were defined as Site1, with K39, Y42, R47, Y57, and D60 identified as candidate hotspots; residues 280–300 of the TF domain were defined as Site2, with D283 and M287 selected as candidate hotspots; Site3 comprised residues 400–423 and 458–481 of the DH domain, with E412, R415, R418, Y419, K422, R472, W475, Y477, E479, and K480 designated as candidate hotspots. Site 1 and Site 3 were combined into one design group, Sites 2 and Site 3 into another. For each group, one to three hotspots were selected within each respective Site to generate combinations for scaffolding.

Protein backbones were then generated using RFdiffusion, with lengths constrained between 20 and 45 amino acids [30]. A monomeric subunit from the ArnA hexameric structure, which exhibited a suitable TF-DH interdomain angle, served as the input template. Based on extensive preliminary trials, optimal hotspot combinations and backbone lengths were determined, resulting in the generation of 300 protein backbones (Site 1 and Site 3) and 100 protein backbones (Site 2 and Site 3).

From these 400 designs, the 50 models with the most favorable geometric complementarity to the target binding site were selected for sequence prediction using ProteinMPNN, generating a total of 5000 sequences [31]. The 20 highest-scoring sequences from each design were then subjected to complex structure prediction using AlphaFold3 with an ArnA monomer [32]. By comparing these predictions with the initial design models, one conformationally rational design was selected.

To further optimize this design, the structure of its N-terminal 30 amino acids was fixed using RFdiffusion, and a new round of large-scale design and screening was performed using the same strategy for selecting hotspots and extending the backbone. This process yielded a design with significantly improved stability. To enhance screening efficiency in subsequent experiments, the same RFdiffusion parameters were maintained, and the N-terminal 30 amino acid sequences were fixed using ProteinMPNN while optimizing the remaining regions. After multiple rounds of generation and screening, three additional optimal designs were identified.

For the design of inhibitory peptides targeting the formation of the ArnA-ArnB complex, an analogous pipeline was employed. First, residues 134–142 and 189–206 within the TF domain of ArnA were defined as Site 1, with K137, R138, E192, R200, D205, and E209 identified as candidate hotspots; concurrently, residues 552–561 and 623–633 of the DH domain were defined as Site 2, with R557, R624, and R628 designated as candidate hotspots. Subsequently, Site 1 and Site 2 were combined into a single design group, and one to three hotspots were selected from each site to generate combinations for backbone scaffolding.

Consistent with the methodology mentioned above, protein backbones were generated using RFdiffusion, with their lengths constrained to between 25 and 65 amino acids. The design was initiated using a monomeric subunit from the ArnA hexamer, which exhibited a suitable TF-DH interdomain angle as the input template. From the resulting designs, the 50 backbone models showing the best geometric complementarity to the target binding site were selected for sequence prediction using ProteinMPNN, yielding a total of 5000 sequences. The top 20 highest-scoring sequences from each design were then subjected to complex structure prediction using AlphaFold3 with an ArnA monomer, culminating in the selection of three optimal designs.

## 3. Results

### 3.1. Significant Enrichment of ArnA DH-TF Domain Fusion Architecture in Gammaproteobacteria

**Figure 1:**
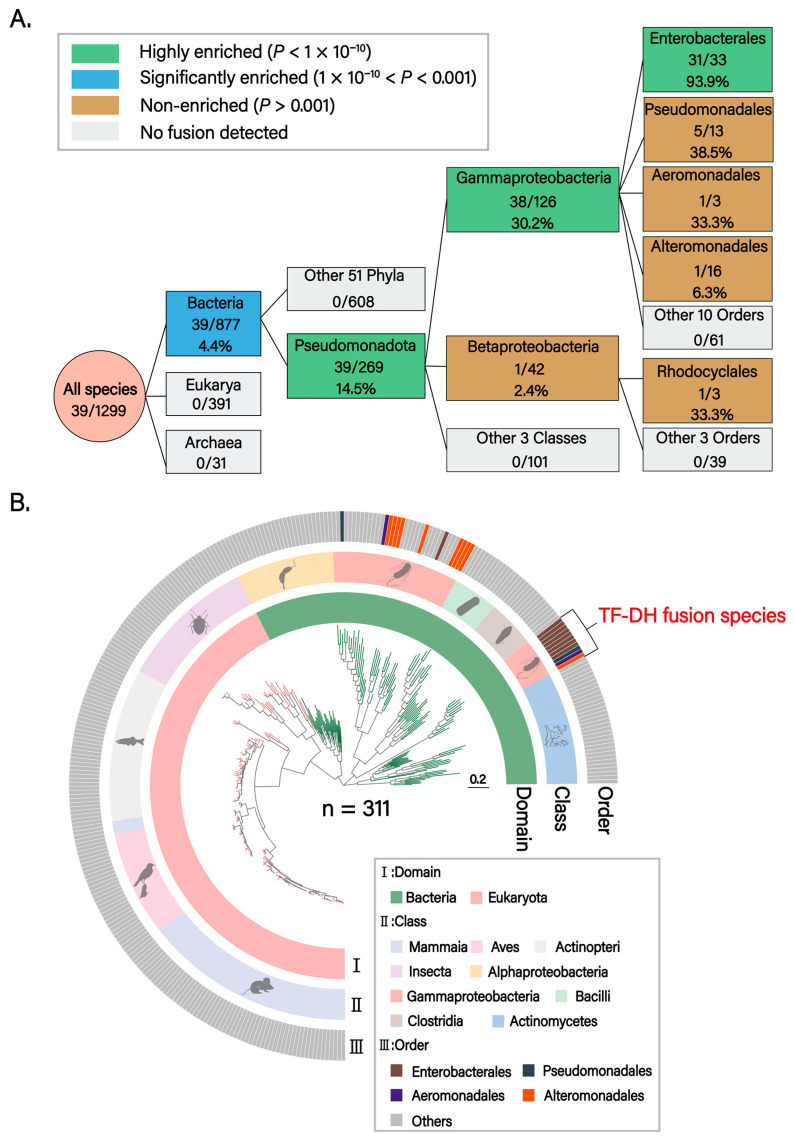
Phylogenetic analysis of TF-DH domain fusion. (**A**) Phylogenetic enrichment analysis of TF-DH domain fusion. The flow diagram illustrates progressive enrichment patterns from the Domain to Order level. Box colors indicate enrichment levels: green (highly enriched, *p* < 1 × 10^−10^), blue (significantly enriched, 1 × 10^−10^ < *p* < 0.001), tan (non-enriched, *p* > 0.001), and gray (no fusion detected). Numbers within boxes show the number of fused species/total species with fusion percentages. Statistical significance was determined by Fisher’s exact test with Bonferroni correction. At each taxonomic level, the target group was compared against all other taxa, excluding itself, to assess enrichment. The analysis reveals a stepwise enrichment trajectory: Bacteria domain (4.4%, *p* = 2.3 × 10^−7^), Pseudomonadota phylum (14.5%, *p* = 1.6 × 10^−11^), Gammaproteobacteria class (30.2%, *p* = 6.3 × 10^−11^), and Enterobacterales order (93.9%, *p* = 7.6 × 10^−12^), indicating strong positive selection for this fusion architecture in these specific lineages. Pseudomonadales order (5/13, 38.5%), despite showing a relatively high fusion rate, did not reach statistical significance compared to the background rate of the other 13 orders (33/113, 29.2%; *p* = 0.53). Betaproteobacteria class (1/42, 2.4%, *p* = 0.015) and other sporadic fusion events lack statistical support. (**B**) Phylogenetic distribution analysis of TF-DH domain fusion. The phylogenetic tree depicts the distribution pattern of TF-DH protein fusion across 311 representative species from 236 genera, 147 families, 77 orders, 9 classes, 5 phyla, and 2 domains. Three concentric rings display taxonomic information at different hierarchical levels: (I) Domain level, showing the distribution of Bacteria (green) and Eukaryota (pink); (II) Class level, highlighting Gammaproteobacteria (pink) as the predominant group harboring fusion proteins; (III) Order level, annotating key orders including Enterobacterales (brown), Pseudomonadales (dark gray), Aeromonadales (purple), and Alteromonadales (orange). The outermost track marks species exhibiting TF-DH domain fusion (red annotation). The phylogenetic tree confirms that TF-DH fusion proteins are predominantly and nearly exclusively present in Gammaproteobacteria, with particular concentration in Enterobacterales, suggesting that this domain fusion represents a lineage-specific evolutionary innovation.

To elucidate the evolutionary origin and distribution pattern of the TF–DH domain fusion in ArnA, we collected proteomes from 2112 eukaryotic, bacterial, and archaeal species for phylogenetic analysis. Following Genome Taxonomy Database (GTDB)–based taxonomic classification and removal of species with incomplete taxonomic information, 1876 species were retained for downstream analyses. Using the dehydrogenase (DH) and formyltransferase (TF) domains of *Escherichia coli* ArnA (P77398) as query sequences, respectively, reciprocal BLASTP searches identified homologs containing both DH and TF domains in 1299 species. Among these, 39 encoded DH–TF fusion proteins, whereas in the remainder, the two domains were encoded as separate proteins. A validation BLAST search using the *E. coli* DH–TF fusion sequence across the 39 candidate species confirmed the initial identifications. Phylogenetic analysis demonstrated that DH-TF domain fusion represents a bacterial-specific feature (39/877, 4.4%), with complete absence in eukaryotes and archaea. This fusion architecture was exclusively confined to the phylum Pseudomonadota (39/269, 14.5%) and exhibited significant enrichment in Gammaproteobacteria (38/126, 30.2%), whereas the single fusion event observed in Betaproteobacteria lacked statistical significance (*P* = 0.015). Within Enterobacterales, domain fusion events approached near-fixation (31/33, 93.9%), indicating robust positive selection for this architectural innovation at this taxonomic level (Figure 1A). The phylogenetic tree of fusion-containing species corroborated this Gammaproteobacteria-specific distribution (Figure 1B).

To further investigate whether DH or TF engages in alternative domain fusion partners, we performed Pfam domain annotation on approximately 14.63 million proteins from 1191 species. A total of 6838 DH or TF homologs were identified; however, apart from DH–TF fusion, no other fusion types, such as DH–X or TF–X, were observed to exist stably in at least three species, indicating that the functional coupling between DH and TF is highly selective and conserved. Although domain separation is more prevalent across most species, the DH–TF fusion in Gammaproteobacteria exhibits enrichment of recurrent positive selection and long-term retention, suggesting that this fusion possesses specific and critical functional necessity within this lineage.

### 3.2. Optimized Cryo-EM Resolves the ArnA Hexamer and Its Interdomain Linker

**Figure 2:**
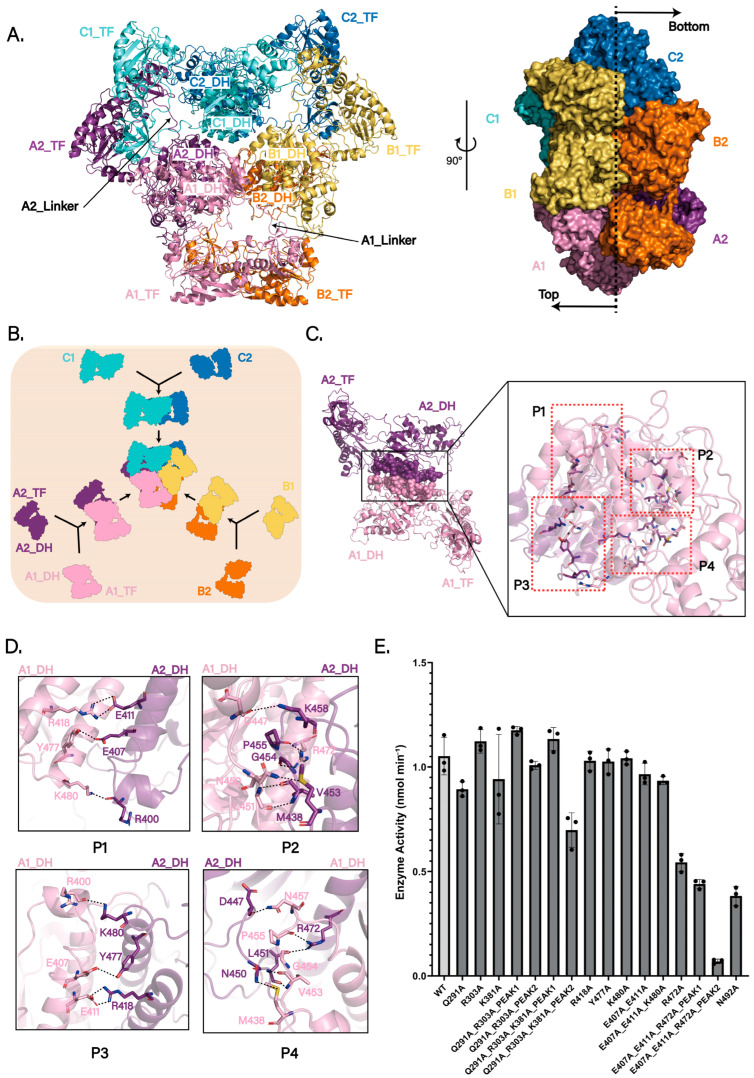
Hexameric assembly and catalytic importance of ArnA. (**A**) Cryo-EM structure of the apo ArnA hexamer (PDB: 9WI0). The six protomers are colored and named according to the three DH-domain-mediated dimeric units: A1 (pink) and A2 (purple); B1 (light orange) and B2 (orange); C1 (aquamarine) and C2 (sky blue) (left panel). Side view (right panel) shows that protomers A1, B1, and C1 localize to one face (top), while A2, B2, and C2 reside on the opposite face (bottom). (**B**) Proposed assembly pathway of the ArnA hexamer as a trimer of DH-mediated dimers (A1–A2, B1–B2, and C1–C2). (**C**) Detailed view of the extensive interaction interface between the DH domains of protomers A1 and A2. Key interacting regions are labeled P1–P4. (**D**) Close-up view of the hydrogen-bonding network (dashed lines) within the A1–A2 DH-domain interface, which stabilizes the dimeric assembly(P1–P4). (**E**) Steady-state kinetics of ArnA dehydrogenase activity monitored at 340 nm. Initial velocities (nmol min^−1^) correspond to NADH formation during the oxidative decarboxylation of UDP-GlcA by NAD+. Activities of wild-type (WT) ArnA and 12 interface mutants are shown. Size-exclusion chromatography (SEC) profiles for three of these mutants (indicated) reveal two distinct peaks: Peak 1 corresponds to the hexameric state, and Peak 2 represents a later-eluting sub-hexameric oligomer. Data are presented as means ± s.d. from three independent replicates. In vitro activity of the active-site mutant N492A, previously shown to abolish catalysis, is included as a negative control.

In light of our bioinformatic findings, we next investigated how TF–DH domain fusion and hexamerization contribute to ArnA catalysis and regulation in *Escherichia coli*. To elucidate the assembly mechanism and resolve potential interdomain dynamics, we determined a high-resolution cryo-electron microscopy (cryo-EM) structure of ArnA (PDB: 9WI0) (Figure 2A). The protein was expressed in *E. coli* BL21 (Rosetta) and purified. In initial experiments, the sample exhibited severe preferred orientation on conventional cryo-EM grids, yielding a reconstruction with a nominal resolution of 3.04 Å but suboptimal map quality. We therefore optimized specimen preparation by employing graphene-coated grids, which mitigated preferred orientation and enabled the collection of a larger dataset of particles. This approach enabled the determination of the apo-ArnA structure at an overall resolution of 2.89 Å, with markedly improved map quality compared to the initial reconstruction (Figure S4A).

The structure confirms a homohexameric organization, consistent with prior studies [19] (Figure 2A). The hexameric core is stabilized by extensive hydrogen-bonding and salt-bridge networks among the six DH domains, which are arranged symmetrically in the center with threefold rotational symmetry. The six TF domains are arranged radially around this core, each tethered to its cognate DH domain via a flexible linker. Importantly, the map resolves the previously unmodeled interdomain linker between the DH and TF domains (residues 304–314) (Figure 2A and Figure S4B). This linker not only physically connects the two domains but also likely facilitates the assembly of hexamers. Furthermore, its flexibility provides a dynamic basis for coupling ArnA’s two catalytic activities in the L-Ara4N biosynthetic pathway.

### 3.3. Hexamerization via DH-Mediated Dimers Is Critical for ArnA Activity

To investigate how hexamer formation contributes to ArnA function, we analyzed interaction patterns in our cryo-EM structure and, in comparison with prior work, propose a more reasonable assembly mode (Figure 2B). Using the chain labels defined above (A1, A2, B1, B2, C1, C2), earlier studies described two alternative hexamer assembly schemes [19,23] (Figure S5A): scheme 1, a dimer of trimers, in which two trimers (A1–B1–C1 and A2–B2–C2) associate to form the hexamer; and scheme 2, a trimer of dimers mediated by TF–TF contacts, comprising the pairs A1–B2, A2–C1, and B1–C2. Using chain A1 as a reference, we analyzed the assembly mode for these models. In scheme 1 (dimer of trimers), hydrogen bonds (A1_TF Arg303 and B1_DH Glu521, A1_DH Lys381 and B1_DH Asp368, A1_DH Arg601 and C1_TF Leu298, A1_DH Glu366 and C1_DH Tyr378) form the A1–B1/C1 interfaces (Figure S5B). In scheme 2 (TF-mediated trimer of dimers), hydrogen bonds (A1_TF Gly53 and B2_TF Gln288, A1_TF Gln291 and B2_TF Val56) form the interface between the TF domains of A1 and B2 (Figure S5C).

In contrast to these arrangements, our structural analysis supports a DH-driven assembly mechanism in which three DH-mediated dimers form first (A1–A2, B1–B2, C1–C2), followed by association of these three dimers into the hexamer, i.e., a trimer of DH-mediated dimers (Figure 2B). In this view, the A1_DH–A2_DH interface exhibits stronger and more extensive interactions than those in either scheme 1 or scheme 2, including hydrogen bonds between A1_DH Arg418 and the backbone of A2_DH Glu411, A1_DH Leu451 and A2_DH Val453, A1_DH Arg472 and A2_DH Pro455, A1_DH Tyr477 and A2_DH Glu407, A1_DH Lys480 and A2_DH Arg400 (Figure 2C,D). These features identify the DH–DH dimer as a structurally robust intermediate, primed for higher-order assembly. From a kinetic perspective, proceeding through this stable dimer offers distinct advantages: it may reduce the activation energy required for hexamer formation, diminish futile collisions, and circumvent kinetic traps associated with the formation of powerful interactions in later stages [33,34]. In line with this mechanism, a structural homology search using the ArnA DH domain as a template revealed that several ArnA homologs (organisms in which DH and TF are encoded on separate proteins) adopt natural homodimers with interfaces closely matching the ArnA DH–DH geometry, including mouse L-threonine dehydrogenase (mTDH; PDB: 4YR9), human UDP-α-D-xylose synthase (hUXS; PDB: 4GLL), maize UDP-glucose 4-epimerase (BZU3; PDB: 7XPQ), human GDP-mannose 4,6-dehydratase (hGMD; PDB: 6GPL), and human UDP-galactose 4-epimerase (hGALE; PDB: 1EK5) [35,36,37,38,39].

To test whether hexameric assembly is required for ArnA catalysis, we performed site-directed mutagenesis of residues within distinct chain–chain interfaces of the hexamer (Figure 2E). Mutations targeting contacts proposed in scheme 1 or scheme 2 had negligible effects on activity. In contrast, alanine substitution of Arg472 in the DH–DH interface (R472A) caused a significant decrease in activity, and the triple mutant ArnA(E407A_E411A_R472A) was nearly inactive. Although most variants remained hexameric after purification, ArnA(R303A_Q291A), ArnA(R303A_Q291A_K381A), and ArnA(E407A_E411A_R472A) each showed two peaks by size-exclusion chromatography (SEC). SDS–PAGE of both peaks revealed intact ArnA, indicating that the later-eluting peak corresponds to a sub-hexameric oligomeric state (Figure S5D). Taken together, these findings support a DH-driven assembly mechanism and suggest that the integrity of the DH–DH interface and the hexameric state are critical for full ArnA activity.

### 3.4. Hexamer Dynamics Coordinate ArnA’s Dual Catalytic Activities

ArnA’s DH and TF domains participate in step 2 and step 4, respectively, of the L-Ara4N biosynthetic pathway, whereas ArnB catalyzes step 3. Previous studies, therefore, proposed that ArnA hexamerization may not only enhance catalytic efficiency but also create a substrate channel for L-Ara4N synthesis [19,23].

**Figure 3:**
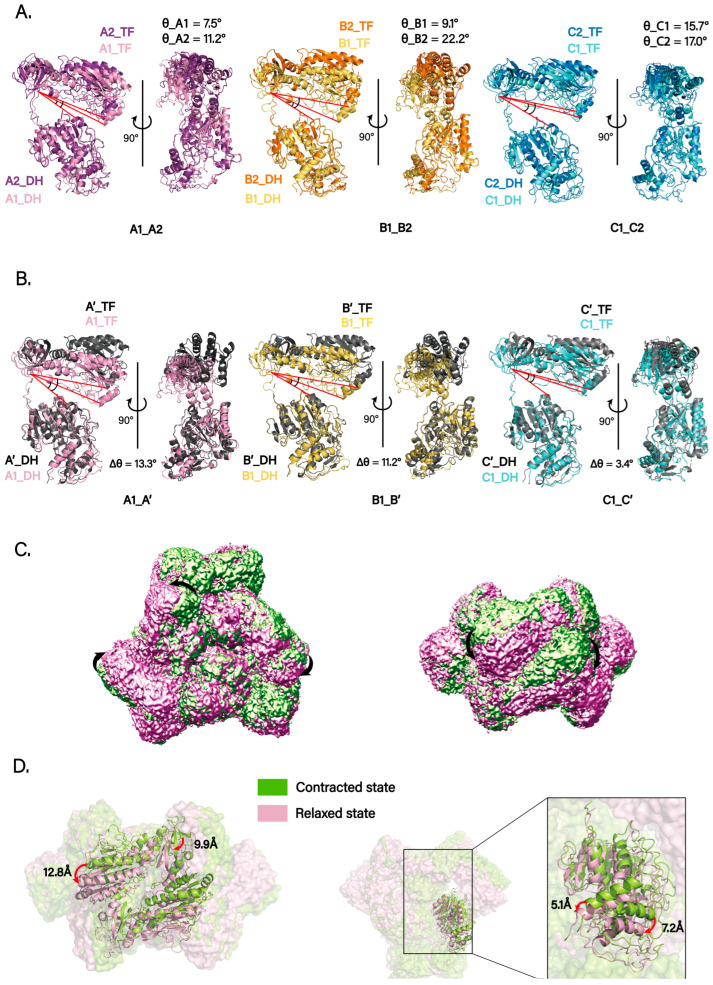
Conformational dynamics of the ArnA hexamer. (**A**) Superposition of the six protomers within the ArnA hexamer, aligned on their DH domains, reveals substantial variability in the interdomain angle (*θ*) between the DH and TF domains. The red lines highlight the interdomain axes, the angle between which defines *θ*. The core of the DH domain is highly conserved and rigid, providing a stable structural framework for this comparative analysis. Each protomer is colored as in Figure 2A, with its measured *θ* value labeled. The systematic difference in *θ* between the two protomers within each DH-mediated dimer (e.g., A1/A2, B1/B2) reveals an intrinsic conformational asymmetry. (**B**) Structural comparison of selected protomers (A1, B1, C1) from the cryo-EM structure with their counterparts from the crystal structure (PDB: 4WKG; shown in gray). These alignments were likewise performed on the DH domains, highlighting the differences in interdomain angles (Δ*θ*). The red lines indicate the interdomain axes for both the cryo-EM and crystal structures, visually emphasizing the angular difference (Δ*θ*). This comparison indicates that the cryo-EM structure adopts a more compact global conformation than the crystal structure. (**C**) A representative trajectory of domain motion derived from 3D variability analysis (3DVA), illustrating the “contract–relax” dynamics of the hexamer. The arrow indicates the direction of domain movement during the conformational transition. (**D**) Two distinct conformational states (contracted and relaxed) were resolved by 3DVA and particle clustering. The models were superimposed by rigid-body fitting, showing the magnitude of domain displacement (DH domain, ~7 Å; TF domain, up to 13 Å).

In our cryo-EM structure, we observed substantial chain-to-chain variability in the angle between the DH and TF domains within each monomer (hereafter *θ*; Figure 3A). The measured interdomain angles were *θ*_A1 = 7.5°, *θ*_A2 = 11.2°, *θ*_B1 = 9.1°, *θ*_B2 = 22.2°, *θ*_C1 = 15.7°, and *θ*_C2 = 17.0°. Notably, chains A1, B1, and C1 lie on one side of the DH-mediated dimer (top), whereas A2, B2, and C2 reside on the opposite side (bottom) (Figure 2B, right). Within each dimer, the DH–TF angle is consistently smaller on one side than on the other, indicating intrinsic conformational asymmetry.

Using the DH domain as the alignment reference, we superposed each chain onto the corresponding chain from the published crystal structure (PDB: 4WKG). The comparisons revealed heterogeneity in DH–TF angles across monomers. The angle differences for three representative chains were Δ*θ*_A1A′ = 13.3°, Δ*θ*_B1B′ = 11.2°, and Δ*θ*_C1C′ = 3.4° (Figure 3B). Interestingly, relative to the crystal structure, the cryo-EM model adopts a more compact overall conformation with smaller DH–TF angles.

We further performed 3D Variability Analysis (3DVA) in cryoSPARC and, together with inspection of the density maps, visualized domain-motion trajectories for individual chains within the hexamer (Video S1). The trajectories indicate substantial conformational flexibility of the TF domain, whereas the DH domain exhibits more limited movement. Only the C-terminal portion of the DH domain could be reliably tracked to move in concert with TF motion. The overall dynamics follow a “contract–relax” cycle (Figure 3C). Particles were then clustered along the principal components from 3DVA, and two distinct classes were reconstructed. Rigid-body fitting and superposition of the models for these two states, designated state A (contracted) and state B (relaxed), showed maximum movement of about 7 Å for the DH domain and up to 13 Å for the TF domain (Figure 3D).

Taken together, these observations suggest that the flexible interdomain linker (residues 304–314) provides the structural basis for the observed heterogeneity and serves as a molecular hinge, enabling interdomain motion and coordinating the movements of the TF domain within the hexameric scaffold. We therefore hypothesize that these dynamics are necessary for ArnA’s dual-catalytic coordination, with TF domain flexibility promoting allosteric coupling to the DH domain and thereby facilitating the efficient relay from UDP-GlcA oxidation (DH activity) to ArnB catalysis and, subsequently, to the formylation of UDP-L-Ara4N (TF activity).

### 3.5. Iterative Protein Design Against ArnA Using RFdiffusion and Structural Validation

To inhibit ArnA catalytic activity by preventing its hexamerization, we employed RFdiffusion to design protein scaffolds for peptide inhibitors targeting the ArnA hexamerization interface. The designed peptides must concurrently bind with high specificity to both the DH-DH and TF-TF interaction interfaces of ArnA involved in its hexamer assembly. To facilitate transport across the Gram-negative outer membrane, we prioritized the design of shorter peptide sequences to enhance delivery efficiency.

**Figure 4:**
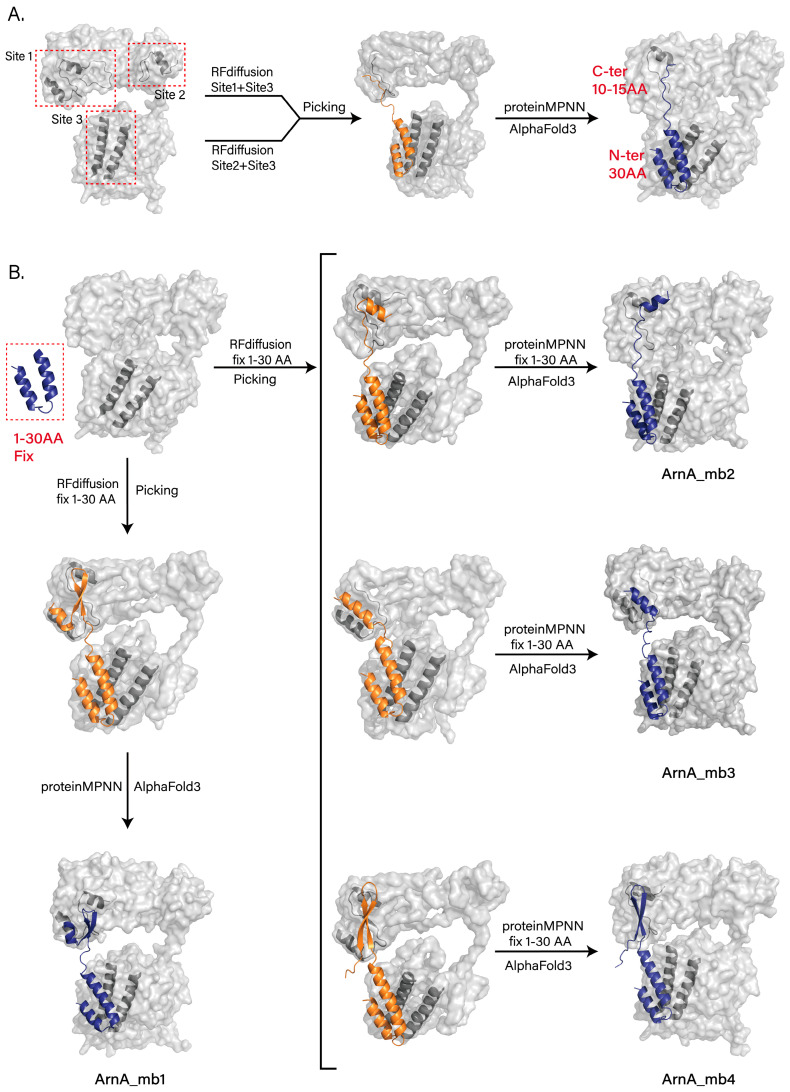
Computational design of peptide inhibitors targeting the ArnA hexamerization interface. (**A**) Schematic of the initial design strategy and representative AlphaFold3-predicted structure from the first design round. RF diffusion-generated peptide designs with two separate hotspot combinations: Site 1 (TF domain) with Site 3 (DH domain), and Site 2 (TF domain) with Site 3 (DH domain). The predicted model shows the stable binding of the N-terminal segment (residues 1−30) to Site 3, while the C-terminal segment (the last 10−15 residues) binds to Site 1 and exhibits conformational flexibility. (**B**) Optimized design pipeline and final peptide models. In the optimized pipeline (Rounds 2 and 3), the N-terminal 30 residues of the top initial designs were fixed. New backbones were then generated by RFdiffusion targeting Site 1, followed by fixed-sequence design using ProteinMPNN and validation with AlphaFold3. This yielded one final design from Round 2 (ArnA_mb1, left) and three additional stable peptides from Round 3 (ArnA_mb2−4, right), all of which achieved the intended binding mode with the N-terminus at Site3 and the structured C-terminus at Site1. All results generated by RFdiffusion are displayed in orange, and all results predicted by AlphaFold3 are shown in deep blue.

We selected two hexamerization interface regions on the TF domain of ArnA (Site 1 and Site 2) and one hexamerization interface region on the DH domain (Site 3) as targets. In the first round of design, we employed RFdiffusion with hotspots defined at Site 1 and Site 3, as well as at Site 2 and Site 3, to generate a large number of backbone designs. From these, we screened the top 50 backbone designs for sequence design using ProteinMPNN. For each design, the top 20 scoring sequences were selected, and their complex structures with ArnA were predicted using AlphaFold3 for validation. Analysis of the predictions revealed that the successfully designed peptides bound stably to Site3 through their N-terminal segments (residues 1–30), whereas the C-terminal segments (last 10–15 residues) bound to Site1 but exhibited conformational flexibility (Figure 4A).

To improve binding stability, we fixed the N-terminal 30 residues from the initial designs in subsequent rounds. We then used RFdiffusion again, this time defining hotspots only at Site1, to generate new backbones. The most promising backbones were selected, sequences were designed using ProteinMPNN, and complex structures were validated with AlphaFold3. This process yielded one final design that bound stably to both Site1 and Site3 (ArnA_mb1) (Figure 4B, left). However, the screening efficiency in this optimization round remained suboptimal, partly because the N-terminal 30 residues were not fixed during the ProteinMPNN sequence design step.

To further enhance efficiency, we refined the screening pipeline. Maintaining the same RFdiffusion backbone generation strategy, we fixed the N-terminal 30-residue sequence during the ProteinMPNN sequence design phase. The resulting designs were re-evaluated and screened using AlphaFold3, ultimately identifying three additional peptides (ArnA_mb2-4), all of which bound stably to both target regions as intended (Figure 4B, right).

### 3.6. Structural Prediction and Inhibitory Design of the ArnA–ArnB Interface

In the L-Ara4N modification, the intermediate UDP-Ara4O, generated by the DH domain of ArnA from UDP-GlcA, is catalyzed by ArnB to form UDP-L-Ara4N [11,13,14,15]. The TF domain of ArnA subsequently formylates this product to yield the final product, UDP-L-Ara4FN. Thus, the two sequential reactions catalyzed by ArnA’s distinct domains require the functional intervention of ArnB.

**Figure 5:**
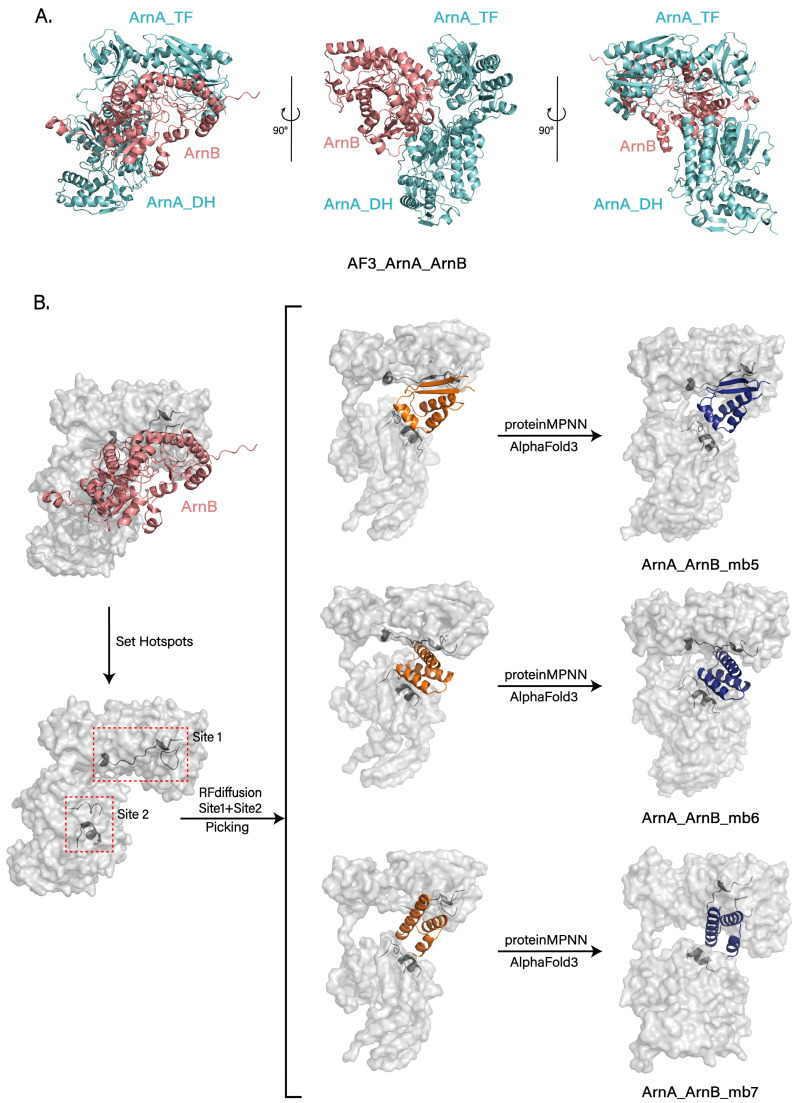
Computational insights into ArnB binding and design of interface-blocking peptides. (**A**) Predicted structure of the ArnA–ArnB complex generated by AlphaFold3. ArnB (soft red) binds on the face opposite the hexameric core and induces a conformational shift in the ArnA TF domain (soft cyan). This movement is consistent with the dynamic flexibility observed in cryo-EM analysis. (**B**) Design of peptides that target the ArnA–ArnB interface. Design workflow involving RFdiffusion with hotspots at two interface sites (Site 1, Site 2), followed by ProteinMPNN sequence design and validation with AlphaFold3. After screening, three results were obtained, effectively blocking the predicted ArnB binding sites (ArnA_ArnB_mb5−7). All results generated by RFdiffusion are displayed in orange, and all results predicted by Alphafold3 are shown in deep blue.

To investigate the interaction mode between ArnA and ArnB, we first predicted the ArnA–ArnB complex structure using AlphaFold3 [32]. The expected model positions ArnB on the face of ArnA opposite the hexameric interaction interface and suggests that its binding induces a conformational shift of the TF domain towards this interface (Figure 5A). This conformational change is consistent with the hinge motion mediated by the linker connecting ArnA_TF and ArnA_DH, as well as the trajectory of the TF domain captured in the 3D variability analysis (Figure 3C).

Further analysis of the interactions between paired TF domains and other monomers within the hexamer revealed only a weak interaction, primarily between Arg303 and Glu521 from a DH domain. This comparatively weak interfacial binding, combined with the inherent dynamic nature of the TF domain, likely provides the necessary space for ArnB binding and could potentially facilitate the formation of a substrate channel for directed transfer of the intermediate between active sites.

To test this hypothesis, we expressed and purified ArnB from *E. coli* BL21 (Rosetta). Incubation of purified ArnB with ArnA, substrate UDP-GlcA, and NAD+ in vitro, however, did not yield a stable complex as assessed by SDS-PAGE and size-exclusion chromatography. Similarly, co-expression and purification of ArnA with ArnB also failed to isolate a complex. Although we were unable to confirm a stable complex under the current experimental conditions, potentially due to specific requirements or the absence of cofactors, these results provide important insights and direction for future mechanistic studies.

Building on our previous design of ArnA_mb1-4 peptides that suppress ArnA hexamerization, we directed our efforts toward the ArnA–ArnB complex to inhibit L-Ara4N synthesis further. Leveraging an AlphaFold-predicted structure of this complex, we employed RFdiffusion to design peptides that target its binding interface and prevent complex formation. To this end, we first identified the key binding epitopes, delineating two primary regions (Site 1 and Site 2). Hotspots within these sites were selected for targeting, and the peptide length was constrained to 25–65 amino acids. Following the method mentioned earlier, we generated a large number of backbones and screened the top 50 designs for sequence design using ProteinMPNN. For each design, the top 20 scoring sequences were selected, and their complex structures with ArnA were predicted using AlphaFold3 for validation. This process ultimately yielded three peptides that stably bind the target regions as designed and effectively occlude the ArnB binding site (ArnA_ArnB_mb5-7) (Figure 5B).

The peptides we designed (ArnA_mb1-4, ArnA_ArnB_mb5-7) have the potential to target both the ArnA hexamerization interface and the ArnA–ArnB binding interface. By inhibiting ArnA oligomerization and complex formation with ArnB, they consequently suppress the production of L-Ara4N. However, this potential remains contingent upon systematic experimental validation and optimization, encompassing structural validation of binding modes and functional evaluation of inhibitory efficacy.

## 4. Discussion

By integrating evolutionary genomics, high-resolution cryo-electron microscopy (cryo-EM), and computational protein design, we systematically elucidate the molecular mechanism of ArnA. Our evolutionary analysis reveals that the DH and TF domains of ArnA exist independently in eukaryotes and most prokaryotes, suggesting that domain separation represents the ancestral state. Notably, the DH-TF fusion event is not randomly distributed but is significantly and specifically enriched in the Gammaproteobacteria class. This suggests that this fusion is a derived feature, acquired independently during the evolution of this taxonomic group. We propose that this domain fusion might optimize metabolic efficiency by enabling substrate channeling or coordinated gene expression, thereby conferring a significant adaptive advantage in specific ecological niches. Furthermore, aside from the DH-TF fusion, no other evolutionarily stable fusion types were identified, further supporting the high specificity of this functional coupling, which is likely driven by unique biological requirements rather than random genomic rearrangements. From an evolutionary perspective, this study elucidates the origin of ArnA’s bifunctional architecture and offers a representative model for domain fusion and functional integration.

Our 2.89 Å cryo-EM structure of apo-ArnA resolves the previously undefined linker (residues 304–314) connecting the DH and TF domains and reveals a DH domain-driven hexamer assembly pathway. Structural and sequence analyses suggest that ArnA assembles via dimer formation followed by trimerization, supported by homologous DH dimers found in other proteins. Functional assays confirm that hexamerization is essential for dehydrogenase activity, highlighting oligomerization as a regulatory feature. 3D variability analysis uncovers marked conformational heterogeneity, especially a “contraction-relaxation” motion of the TF domains around the flexible linker. This dynamic motion likely coordinates the sequential catalytic steps by enabling efficient substrate channeling between the two domains. The observed asymmetry across dimers may act as a built-in regulatory mechanism for temporal control of catalysis.

Leveraging these structural insights, we developed a structure-guided peptide design strategy targeting the hexamerization interface and inter-domain flexibility. Designed peptides bind to intermediate assembly states or restrict TF mobility, potentially disrupting ArnA function and L-Ara4N biosynthesis. This approach provides a novel angle for combating polymyxin resistance. In addition, AlphaFold3 modeling suggests a plausible interaction between ArnA and ArnB, mediated by the flexible TF domains. Although not experimentally confirmed, this interface may facilitate substrate channeling across enzymes. We further designed peptides targeting the predicted ArnA–ArnB interface, offering a potential means to block this interaction and downstream modification steps.

This study outlines clear directions for future work. Determining the cryo-EM structures of ArnA in complex with its substrates or ArnB will be critical for elucidating the structural basis for substrate channeling and sequential catalysis. Furthermore, the designed peptides targeting the hexamerization interface or the predicted ArnA–ArnB interface represent high-potential candidates whose efficacy awaits experimental validation through in vitro biophysical and functional assays. Such investigations will not only deepen the mechanistic understanding of ArnA but also accelerate the development of novel therapeutic strategies against polymyxin resistance.

## 5. Conclusions

Our integrated analysis reveals the molecular basis of ArnA’s bifunctionality and its role in polymyxin resistance. We demonstrate that DH–TF domain fusion, enriched in Gammaproteobacteria, reflects an evolutionary adaptation to enhance catalytic coordination. The cryo-EM structure uncovers a DH-driven hexameric assembly essential for activity, and conformational dynamics likely mediate domain interplay and potential substrate channeling with ArnB. Guided by these insights, we designed peptide inhibitors targeting key interfaces of ArnA, offering new leads for therapeutic development. Future work should validate these candidates and resolve the ArnA–ArnB complex to further illuminate this resistance pathway.

## Data Availability

The atomic coordinates have been deposited in the Protein Data Bank, www.rcsb.org, accessed on 1 December 2025, under accession code 9WI0. The cryo-EM maps have been deposited in the Electron Microscopy Data Bank under accession codes EMD-65981.

## Supplementary Materials

The following supporting information can be downloaded at: https://www.mdpi.com/article/10.3390/biom15111594/s1, Figure S1: The biosynthetic pathway of L-Ara4N. Figure S2: Flow chart for cryo-EM data processing of the ArnA without G.O. Data processing details are provided in the Methods section. Figure S3: Flow chart for cryo-EM data processing of the ArnA with G.O. Data processing details are provided in the Methods section. Figure S4: Refinement of the Density Map and Determination of a Previously Unresolved Linker Region. (A) The initial cryo-EM map suffered from severe preferred orientation (teal green). This issue was mitigated by data collection using graphene-coated grids (muted purple). (B) The map enabled the resolution of the previously unresolved interdomain linker (residues 304–314) connecting the DH and TF domains. Shown is the linker region fitted into the density map for two protomers, A1 (pink) and A2 (violet purple). Figure S5: Assessment of proposed ArnA hexamer assembly models. (A) Schematic overview of two alternative hexamer assembly schemes from prior studies. Scheme 1 depicts a “dimer of trimers,” where two trimers (A1–B1–C1 and A2–B2–C2) associate (left panel). Scheme 2 depicts a “trimer of dimers” mediated primarily by TF–TF domain contacts, forming the pairs A1–B2, A2–C1, and B1–C2 (right panel). (B) Detailed molecular interactions proposed for Scheme 1 (dimer of trimers). The interface between protomer A1 and its neighbors (B1, C1) is stabilized by a network of hydrogen bonds (using chain A1 as a reference). (C) Detailed molecular interactions proposed for Scheme 2 (TF-mediated trimer of dimers). The interface is shown for the A1–B2 dimer pair, stabilized by hydrogen bonds between the TF domains (using chain A1 as a reference). Video S1: Domain-Motion Trajectories within the ArnA Hexamer. (D) Unlike the wild-type hexamer, the ArnA(R303A_Q291A), ArnA(R303A_Q291A_K381A), and ArnA(E407A_E411A_R472A) mutants each exhibit two peaks by size-exclusion chromatography (SEC). SDS-PAGE analysis confirms that both peaks contain intact ArnA protein, indicating that the later-eluting peak corresponds to a sub-hexameric oligomeric state.
