# Supplementary figures and images for: Structural Basis for Targeting the Bifunctional Enzyme ArnA
Source: Biomolecules. 2025 Nov 13;15(11):1594. doi: 10.3390/biom15111594 (PMC12650200; doi:10.3390/biom15111594)

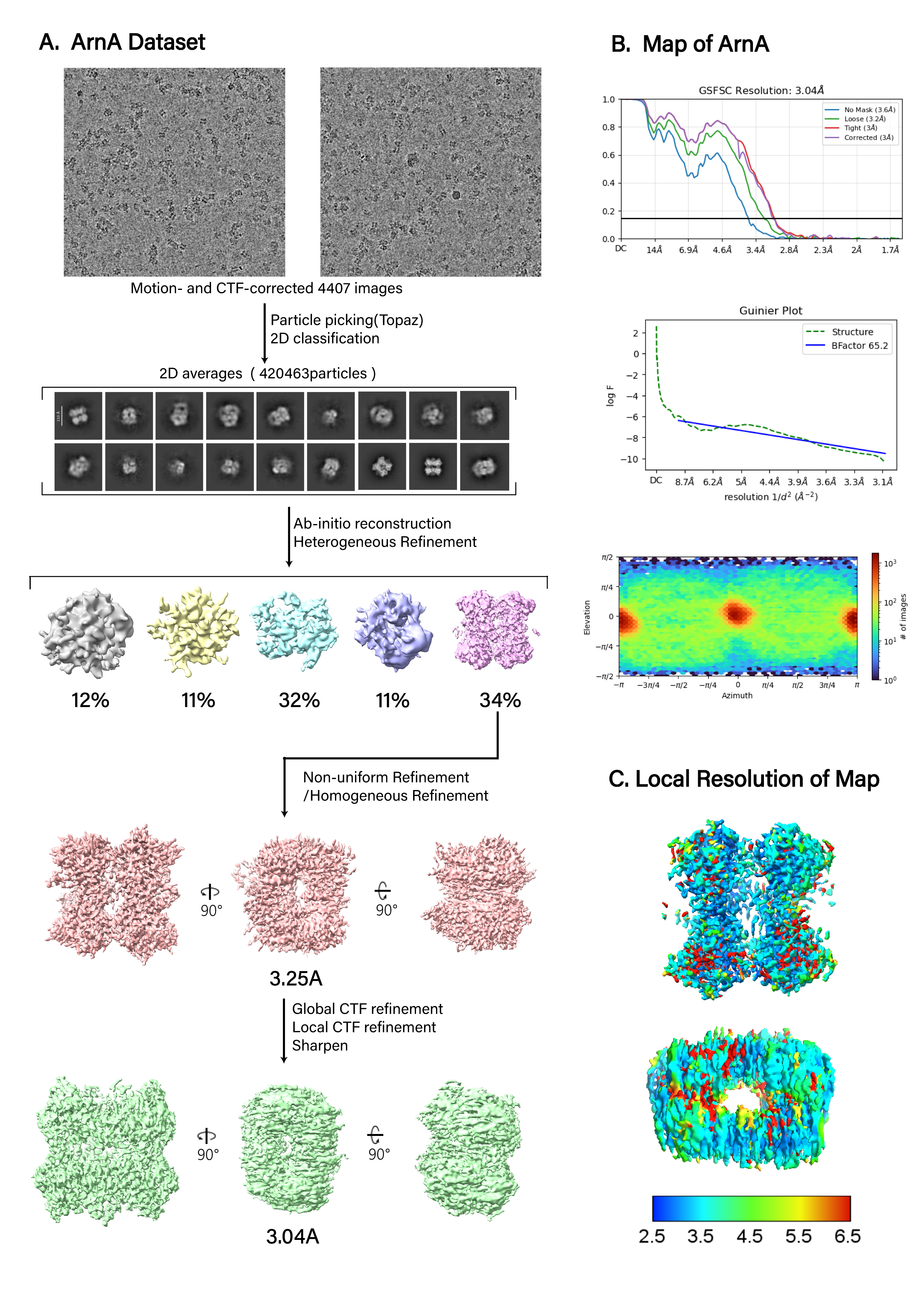

Supplement: Supplementary file 1 [file biomolecules-15-01594-s001.zip › biomolecules-3967856-supplementary-update/Figure_S2.tif]

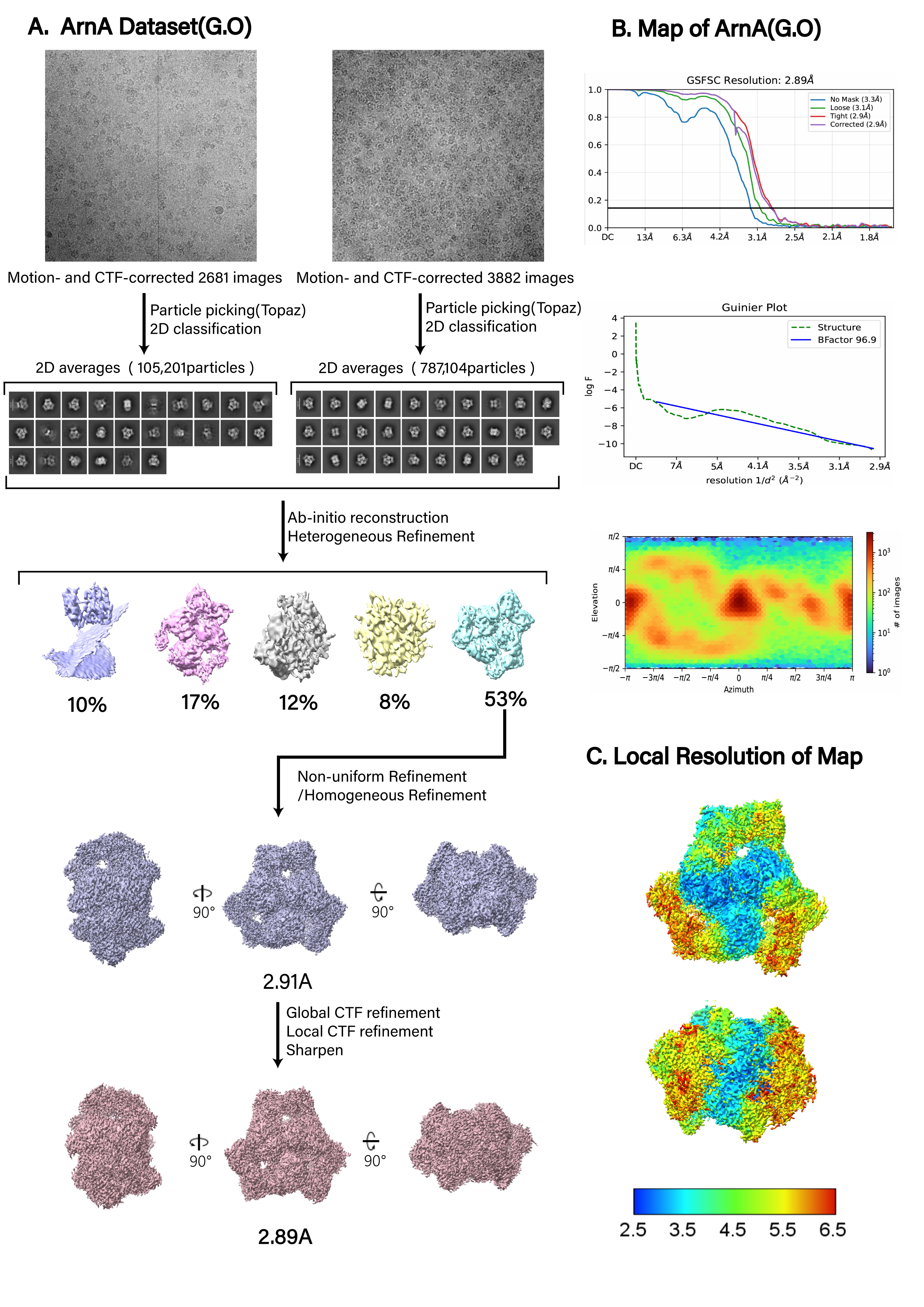

Supplement: Supplementary file 1 [file biomolecules-15-01594-s001.zip › biomolecules-3967856-supplementary-update/Figure_S3.tif]
